# Cooperation of Nutlin-3a and a Wip1 inhibitor to induce p53 activity

**DOI:** 10.18632/oncotarget.9302

**Published:** 2016-05-11

**Authors:** Anusha Sriraman, Marija Radovanovic, Magdalena Wienken, Zeynab Najafova, Yizhu Li, Matthias Dobbelstein

**Affiliations:** ^1^ Institute of Molecular Oncology, Göttingen Center of Molecular Biosciences (GZMB), University Medical Center Göttingen, Göttingen, Germany; ^2^ Department of General, Visceral, and Pediatric Surgery, University Medical Center Göttingen, Göttingen, Germany

**Keywords:** Mdm2, Wip1/PPM1D, p53, kinase, phosphatase

## Abstract

Targeting the Mdm2 oncoprotein by drugs has the potential of re-establishing p53 function and tumor suppression. However, Mdm2-antagonizing drug candidates, e. g. Nutlin-3a, often fail to abolish cancer cell growth sustainably. To overcome these limitations, we inhibited Mdm2 and simultaneously a second negative regulator of p53, the phosphatase Wip1/PPM1D. When combining Nutlin-3a with the Wip1 inhibitor GSK2830371 in the treatment of p53-proficient but not p53-deficient cells, we observed enhanced phosphorylation (Ser 15) and acetylation (Lys 382) of p53, increased expression of p53 target gene products, and synergistic inhibition of cell proliferation. Surprisingly, when testing the two compounds individually, largely distinct sets of genes were induced, as revealed by deep sequencing analysis of RNA. In contrast, the combination of both drugs led to an expression signature that largely comprised that of Nutlin-3a alone. Moreover, the combination of drugs, or the combination of Nutlin-3a with Wip1-depletion by siRNA, activated p53-responsive genes to a greater extent than either of the compounds alone. Simultaneous inhibition of Mdm2 and Wip1 enhanced cell senescence and G2/M accumulation. Taken together, the inhibition of Wip1 might fortify p53-mediated tumor suppression by Mdm2 antagonists.

## INTRODUCTION

The tumor suppressor p53 is mutant in roughly 50% of all human malignancies, making it the most frequently mutated gene in human cancers. However, this notion also implies that another 50% of cancers still carry wild type p53 and nonetheless become malignant. In these cases, the tumor-suppressive activity of p53 is attenuated by regulatory mechanisms [1].

The best-characterized activity of p53 consists in transcriptional activation, through binding to its cognate promoter elements and recruiting transcription initiation factors as well as chromatin modifiers. This activity can be induced by cell stress signaling events, through a cascade of phosphorylations and acetylations. The p53-responsive genes and their products induce cell cycle arrest and/or senescence (e. g. p21/CDKN1A), or apoptosis (e. g. Puma/BBC3). A third set of p53-inducible genes provides negative feedback on p53 activity, thereby attenuating the initial p53 response.

The expression of the negative regulator Mdm2 can be induced by p53. The Mdm2 protein binds to p53 and interferes with transactivation. Moreover, Mdm2 is an E3 ubiquitin ligase, leading to p53 ubiquitination and proteasomal destabilization. The Mdm2 gene is amplified in a considerable proportion of malignant tumors, most notably in sarcomas. In these cases, excessive amounts of Mdm2 largely abolish the tumor suppressive activity of p53. However, even when the copy number of the Mdm2 gene is normal, the p53-antagonizing activity of Mdm2 can still be exaggerated in tumors. The most well-established mechanisms for this consist in the silencing of a negative regulator of Mdm2, p14ARF (the second product of the gene CDKN2A), or in the enhanced expression of the heterodimerization partner of Mdm2, MdmX/Mdm4 [2].

Given the frequent silencing of p53 by the Mdm2 oncoprotein in tumors, it is conceivable that disrupting the interaction between the two proteins might re-establish p53-mediated tumor suppression [3]. Furthermore, the p53-binding structure on Mdm2, a hydrophobic pocket domain, can be occupied by a small molecule, making this one of the earliest example of “drugging” a protein-protein interaction. The most established compound to achieve this is Nutlin-3a [4], shortly referred to as Nutlin from here on. Nutlin binds to Mdm2, competing with p53. As a consequence, p53 becomes more active as a transcription factor and accumulates as a relatively stable protein, due to the lack of ubiquitination by Mdm2 [4].

Most cells respond to Nutlin largely by a reversible cell cycle arrest. Only a few cell lines - the majority of which containing heavily amplified Mdm2 - respond with apoptosis, thus rendering the drug efficient in cell killing. In the meantime, a number of drug candidates with similar structure and/or activity as Nutlin have been developed and have entered clinical trials [3, 5]. A major concern in these trials consists in a possible lack of efficacy. Since many tumor cells merely arrest in response to Nutlin but resume proliferation once the drug is taken off, the clinical response might be transient at best. One way to get around this problem is to select tumors with a high frequency of Mdm2 amplifications, e. g. dedifferentiated liposarcomas [6, 7]. Another way to fortify the efficacy of Nutlin and related drugs would be to combine them with additional compounds. This require targets that, when inactivated along with Mdm2, trigger an additive or even synergistic response.

Besides Mdm2, at least one additional p53-responsive gene product antagonizes p53 activity. The gene Wip1/PPM1D, originally named after its plant homologue “wound-induced protein” [8], is induced by p53 [9]. Its product is a phosphatase that dephosphorylates a variety of proteins that are substrates to the kinase Ataxia Telangiectasia Mutated (ATM) [10] or related DNA damage-responsive pathways, e. g. ATM itself, Chk2 [11, 12], Chk1 [13] Histone 2AX [14-16], Mdm2 [17] and p53 [13, 18]. The phosphorylation of p53 near its aminoterminus (e. g. at the residues Ser15 and Ser20) facilitates the association of acetyl transferases (e. g. p300 and CBP) with p53 and the subsequent acetylation of carboxyterminal residues [19]. This in turn enhances the binding of p53 to its target promoters. The phosphorylated aminoterminal domain then activates transcription, rendering p53 phosphorylation an essential trigger for gene activation [19]. When p53 induces Wip1, the dephosphorylation of p53 counteracts this activity and thus provides a negative feedback, similar to Mdm2. Like Mdm2, Wip1 can drive malignancy. This can happen through gene amplification, but also by truncating mutations near the 3′ end of the coding region, destroying a negative regulatory domain of the Wip1 protein [20-24]. Most Wip1/PPM1D-amplified tumors harbor wild type p53, further arguing that the inactivation of p53 is at least one of the major activities of Wip1 [25].

Specific Wip1 inhibitors have recently been designed. In particular, the drug candidate GSK2830371 was shown to efficiently and specifically interfere with the phosphatase activity of Wip1 through allosteric inhibition [26]. These inhibitors increase the phosphorylation of Wip1 substrates, including p53, and lead to a moderate increase in the expression of some p53 target genes. However, the cytotoxic efficacy of the inhibitors seemed moderate [26].

Of note, Mdm2 and Wip1 are acting by largely independent mechanisms. While Mdm2 triggers the degradation of p53 through the proteasome, Wip1 dephosphorylates its transactivating domain. This argues that inhibiting just one of these antagonists may be insufficient for full p53 activation. Instead, it would be more plausible to boost p53 activity by targeting both of these major antagonists simultaneously. In fact, the depletion or inactivation of both Wip1 and Mdm2 yields strong p53 activity [27-30].

Here we show that the combined inhibition of Mdm2 and Wip1 indeed abolishes cell proliferation in a synergistic and sustainable fashion. When applied together, Nutlin-3a and GSK2830371 induce strong accumulation of phosphorylated and acetylated p53. They also induce the accumulation of p53 target gene products in a p53-dependent fashion. Importantly, the genes induced by Mdm2 inhibition *vs*. Wip1 inhibition alone were largely distinct. Combining both drugs, however, primarily activated a large set of p53-responsive genes. Many of these genes were induced to a greater extent by the combination, rather than by Nutlin alone. Taken together, inhibiting Mdm2 and Wip1 simultaneously may represent a viable strategy to achieve strong p53 activation and permanent growth arrest, thereby diminishing or even preventing tumor progression.

## RESULTS

### Combined inhibition of Mdm2 and Wip1 synergistically diminishes cell proliferation

**Figure 1:**
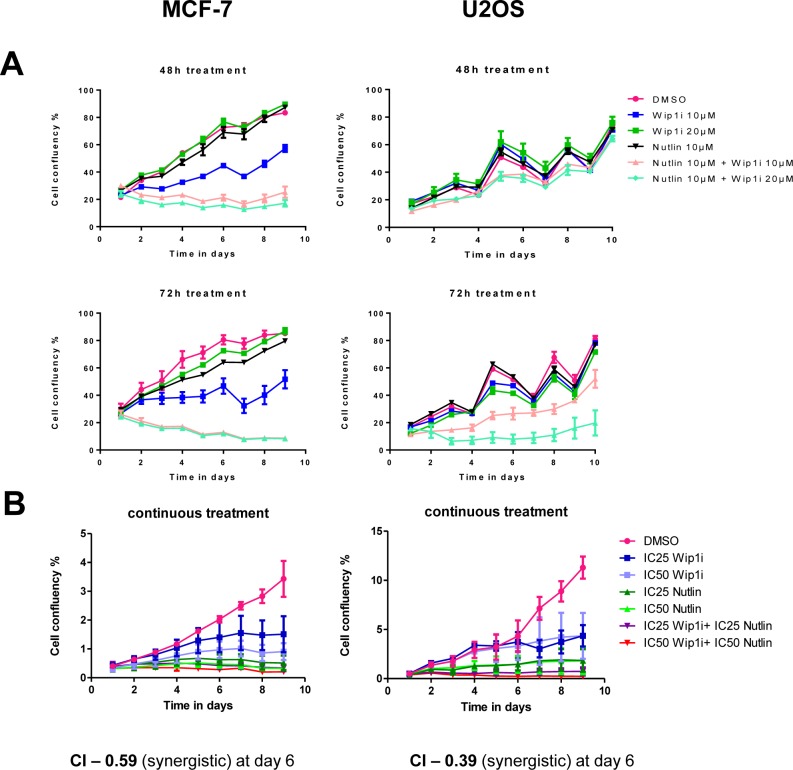
Synergistic impairment of cell proliferation by inhibition of Mdm2 and Wip1 **A.** Co-treatment by Wip1i and Nutlin impedes cell growth in MCF-7 and U2OS cells. MCF-7 and U2OS cells were treated with Nutlin-3a (Nutlin), GSK2830371 (Wip1i) and its combinations at the indicated concentrations. After 48h and 72h of treatment, the drugs were removed and fresh medium was added. Cell confluency was measured every day using bright field microscopy (Celigo cell cytometer). Media was changed every 2-3 days, explaining the fluctuations in cell proliferation. **B.** Synergistic activity of Nutlin and Wip1i on MCF-7 and U2OS cells. MCF-7 and U2OS cells were treated with Nutlin and Wip1i at their IC25 and IC50, respectively, with continuous incubation. The cell confluency was measured daily as in A. Using the Chou-Talalay method [31], the combination index (CI) was calculated. At day 6, strong synergism was reflected by CI values way below 1.

We tested the efficacy and sustainability of treatment with the Mdm2 inhibitor Nutlin-3a (Nutlin), the Wip1/PPM1D inhibitor GSK2830371 (Wip1i), and their combination. The cell lines chosen for this study were MCF-7 (breast carcinoma) and U2OS (osteosarcoma), based on their known amplification (MCF-7) [23] or activating truncation (U2OS) [20] of Wip1. After treating the cells for 48 or 72 h, the drugs were removed and the cells were continuously incubated for ten days. Cell proliferation was followed by automated translucent microscopy. Both drugs were used at concentrations known to increase p53 levels or to enhance the phosphorylation of ATM substrates (Supplemental Figure S1). Nonetheless, neither of the drugs prevented cell proliferation over this period of time, although Wip1 inhibition did slow down the growth rate to some extent. In contrast, the combination of both inhibitors profoundly compromised the outgrowth of both cell lines and prevented confluency over the entire duration of the experiment, with the MCF-7 cells being particularly responsive (Figure 1A). Thus, the two drugs did cooperate to abolish cancer cell proliferation in a sustainable fashion.

To determine whether the two compounds act in a formally synergistic way, we first determined the drug concentrations that reduce cell proliferation by 25% or 50% (IC25 and IC50, respectively). The drugs were then continuously applied to the cells at IC25 and IC50, alone or in combination, and the reduction in proliferation was determined six days after drug removal (Figure 1B). This allowed us to calculate the combination index (CI) according to the algorithm by Chou and Talalay [31]. As a result, CIs far below 1 were obtained, indicating strong synergism between the drugs. Thus, inhibition of Mdm2 and Wip1 not only add up to impair cell growth, but they truly synergize to provide permanent growth arrest.

### In combination with Wip1 inhibitor, Nutlin induces the accumulation of phosphorylated and acetylated p53

**Figure 2:**
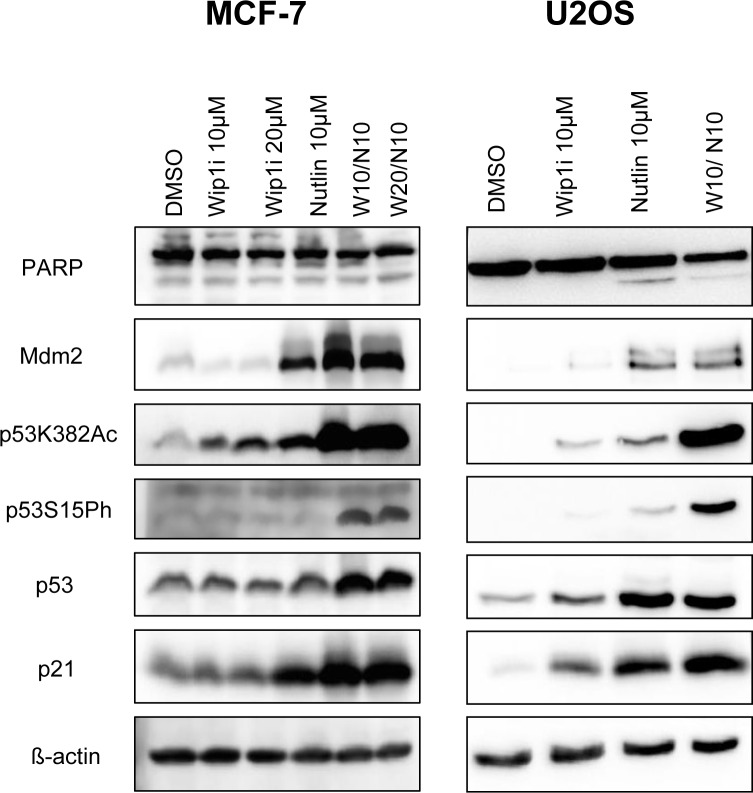
Accumulation of phosphorylated and acetylated p53 upon combined treatment **A.** MCF-7 and U2OS cells were treated with Nutlin and Wip1i as indicated. After 24 h, immunoblot analysis was performed to assay for the activation of p53 - phospho p53 and acetylated p53 - and its target gene products p21 and Mdm2. Actin staining served as the loading control.

To elucidate the mechanism of action underlying this drug synergism, we assessed the levels and modifications of p53, as well as the levels of p53-inducible gene products by immunoblot analysis. Wip1 inhibition alone did not detectably affect the activity of p53 in MCF-7 cells, whereas in U2OS cells, it mildly increased the phosphorylation of p53 at serine 15 and its acetylation at lysine 382. Correspondingly, the p53 target gene products p21 and Mdm2 were somewhat increased by Wip1 inhibition in U2OS cells but not in MCF-7 cells. Nutlin alone increased the levels of p53 and its target gene products in both cell lines. Importantly, when both drugs were combined, modified p53 strongly accumulated, along with p21 and Mdm2 (Figure 2). Of note, none of the treatments led to substantial increases in the cleavage of poly-(ADP ribose) polymerase (PARP), strongly suggesting that the observed reduction in cell numbers (cf. Figure 1) was not primarily a result of apoptosis. Along the same line, caspase activity was not induced by Nutlin or the combination of Nutlin with Wip1i (Supplemental Figure S2). In conclusion, Wip1 inhibition and Mdm2 inhibition cooperate to increase the phosphorylation and acetylation of p53, and to enhance the expression of the p53 target genes p21 and Mdm2.

### The induction of p21 and Mdm2, and growth inhibition by the drug combination, depend on p53

**Figure 3:**
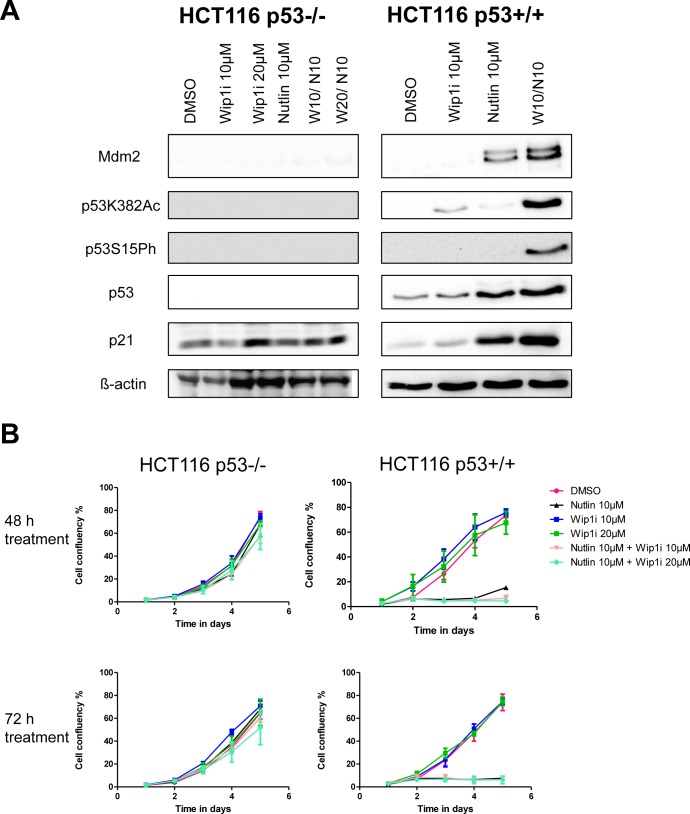
Activation of the p53 response upon co-treatment with Nutlin and Wip1i, dependent on p53 **A.** Co-treatment with Nutlin and Wip1i leads to a p53 dependent response. An isogenic pair of HCT116 cells with and without a targeted deletion of TP53 [32] was treated with Nutlin and Wip1i as indicated. After 24 h, the cells were harvested to prepare protein lysates. Immunoblot analysis was performed to determine the amounts of p53 and its target gene products. **B.** Decrease in cell proliferation upon combined treatment is dependent on p53. Confluency for HCT116 cells with or without p53 was monitored for 5 days. The cells were treated with DMSO, Nutlin, Wip1i and their combination for 48h and 72h. Then, fresh medium was added and cell proliferation was monitored.

Next, we tested whether the inhibitors of Wip1 and Mdm2 are increasing p21 and Mdm2 levels in a p53-dependent fashion. To this end, we treated HCT116 cells (colon carcinoma, wild type p53, activating truncation of Wip1 [20]) or an HCT116-derived cell line with targeted disruption of the p53-encoding genes [32] with the same drugs, alone or in combination. As expected, we observed the accumulation of p21 and Mdm2 only in the p53-proficient cells (Figure 3A), thereby largely excluding off-target effects or any other p53-independent effects of Wip1 and Mdm2 inhibition. In HCT116 cells again, the two drugs cooperated to induce the accumulation of phosphorylated and acetylated p53, and to enhance the expression of p53 target genes. Furthermore, we monitored the proliferation of HCT116 cells with and without p53 in response to the drugs. Reduced cell proliferation upon treatment with Nutlin and/or Wip1i was only observed in p53-proficient cells but not when p53 was deleted (Figure 3B). Thus, the efficacy of Nutlin and Wip1i strictly depends on p53. Finally, we tested whether the knockdown of Wip1 could mimic Wip1 inhibition. We first depleted Wip1 from U2OS cells by siRNA and then monitored the expression of p53-responsive genes by quantitative RT-PCR, in the presence or absence of Nutlin. Indeed, the depletion of Wip1 increased the ability of Nutlin to augment the expression levels of p21, PUMA and PIG3 (Supplemental Figure S3A-S3D). Furthermore, when depleting Wip1 by siRNA, Nutlin compromised cell proliferation to a greater extent than upon control transfection (Supplemental Figure S3E). Thus, Wip1 depletion largely phenocopies Wip1 inhibition when combined with an Mdm2 inhibitor, strongly suggesting that the cooperation of Nutlin and Wip1i actually depends on targeting Wip1.

### While each compound induces distinct gene sets, the combination largely enhances the gene signature of Nutlin

**Figure 4:**
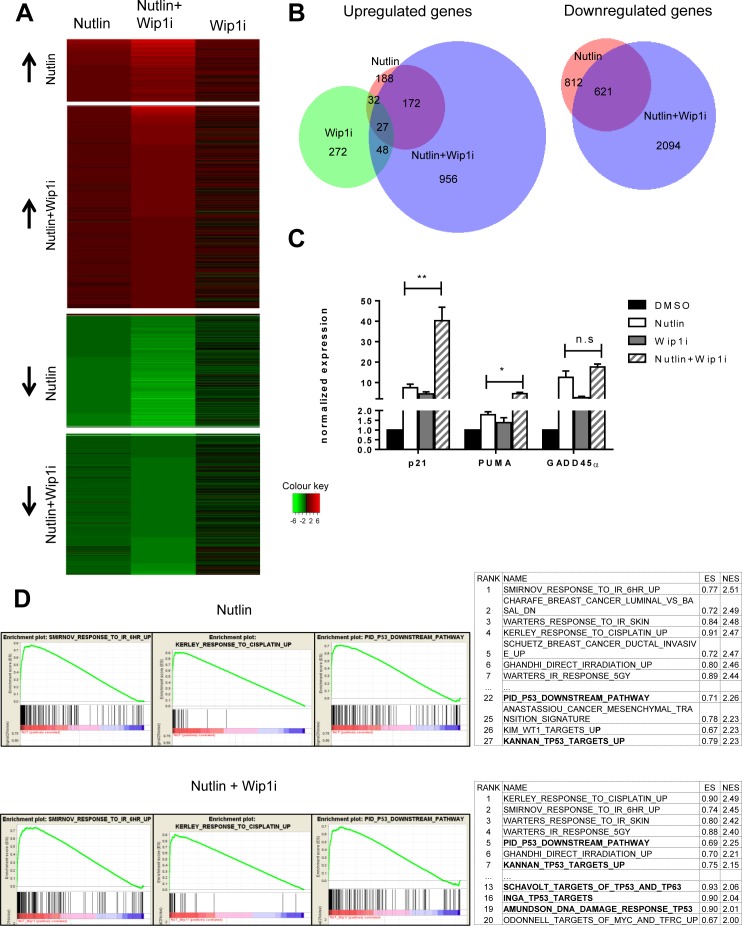
Broadened p53 response upon combined drug treatment, but not upon Wip1 inhibition alone **A.** Heat map with log2-fold changes, resulting from mRNA-sequencing analysis of MCF-7 cells. Nutlin denotes cells treated with Nutlin at 10μM for 16 h compared to DMSO treatment. Wip1i denotes cells treated with GSK 2830371 at 10μM, and Nutlin+Wip1i denotes cells treated with both drugs at the same concentrations, all for 16 h and compared to DMSO treatment. Only genes with Iog2-fold change ≥ 0.85 and an adjusted p-value ≤ 0.05 were included into the heat map. The number of differentially regulated genes under each condition were Nutlin - 474, Wip1i - 272, Nutlin+Wip1i - 1853. For single genes, cf. Supplemental Table 1. **B.** Venn diagrams depicting the significantly downregulated and upregulated genes in MCF-7 cells. Using the Bio-Venn software (www.cmbi.ru.nl/cdd/biovenn/index.php), the significantly upregulated and downregulated genes were plotted under each condition - Nutlin, Wip1i and Nutlin+Wip1i, each *vs*. DMSO. The Iog2-fold change was ≥ 0.85 for Nutlin, Wip1i, and Nutlin+Wip1i, and the adjusted p-value was ≤ 0.05 for each sector. the corresponding numbers of genes are indicated. **C.** Enhancement of p53-induced mRNA synthesis by combined inhibition of Wip1 and Mdm2. MCF-7 cells were treated with the indicated combinations of inhibitors, followed by RNA preparation after 16 h. Gene expression was quantified by real-time RT-PCR (mean±SEM, *n* = 3). **D.** GO term analysis and functional annotation. Gene set enrichment analysis (GSEA) from C2 curated gene sets (provided by the Molecular Signatures Database (MSigDB) v5.0 [60, 61]) was performed using variance stabilized RNA-Seq reads from Nutlin and Nutlin+Wip1i treated samples. Selected enrichment plots from gene sets induced by Nutlin and Nutlin+Wip1i are provided as examples. Ranking tables for induced gene sets are provided to demonstrate increased appearance of p53-responsive gene sets in the combination treatment compared to single treatment with Nutlin.

To obtain a comprehensive overview on the genes induced by each of the drugs and their combination, we performed deep sequencing analysis of the RNA obtained from MCF-7 cells after treatment. As expected, large numbers of genes were found significantly regulated by each treatment (Figure 4A, Supplemental Table 1). Surprisingly, the overlap between genes that were upregulated by Nutlin and by Wip1 inhibition was very limited, comprising only 7% of the Nutlin-responsive genes. Thus, Wip1 inhibition alone induces only a small subset of p53-responsive genes, including CDKN1A, FAS, and Mdm2. Otherwise, it appears to regulate genes by other means, e. g. through the phosphorylation of signaling factors that ultimately affect gene expression. Even more strikingly, however, the combination of Nutlin and Wip1i elicited a gene expression signature that was far closer to Nutlin alone than to Wip1i alone. The combination largely recapitulated the genes induced by Nutlin alone but enhanced their number by inducing additional genes, most of which had not been found inducible by either drug alone. In addition, gene induction was enhanced for a lot of Nutlin-responsive genes in the additional presence of Wip1i. Similarly, while Wip1i did not suppress the expression levels of any gene to a significant extent, Nutlin induced downregulation of p53-repressed genes. The mechanism of p53-mediated gene repression involves the CDK inhibitor p21 and a repressive complex, as described previously [33-36]. Importantly, co-treatment with Wip1i and Nutlin led to the repression of a broader set of genes which included virtually all the genes that had been repressed by Nutlin alone, again supporting the view that the combination broadens and intensifies p53 activity (Figure 4B), and in agreement with previous studies on the role of Wip1 in the G2-M checkpoint [37].

Next, we sought to determine the induction of p53-responsive genes in a more quantitative fashion, after treatment of MCF-7 cells with each or both of the two drugs. When analyzing mRNA levels by quantitative RT-PCR, we found that the combination of both drugs can induce p53-responsive genes up to 50-fold, whereas single drugs never exceeded 10-fold (Figure 4C). Thus, combining both drugs leads to a far more efficient induction of p53 activity than either compound alone. Of note, this degree of induction was greater than what was expected based on the immunoblot analysis shown in Figures 2 and 3. This may be due to the destabilization of p21 [38] and Mdm2 [39] proteins (but not mRNA) through the DNA damage response signaling elicited by Wip1 inhibition. Nonetheless, based on the plethora of additional p53-responsive genes induced, we propose that the boost in p53 activity through the combination of both inhibitors provides an explanation for the sustained growth arrest of p53-proficient cells observed in Figure 1.

Finally, gene set enrichment analysis (GSEA) revealed that “irradiation” and “p53 signaling pathway” were by far the most significantly enriched terms associated with the genes induced by the simultaneous treatment with Nutlin and Wip1i (Figure 4D; Supplemental Table 2). Remarkably, gene sets regarding p53 signaling pathways were induced to a much greater extent in the combination treatment in comparison to the Nutlin treatment alone, as indicated by GSEA term ranking.

### Nutlin and the combination of Nutlin with Wip1i preferentially induce genes with promoters that physically bind p53

**Figure 5:**
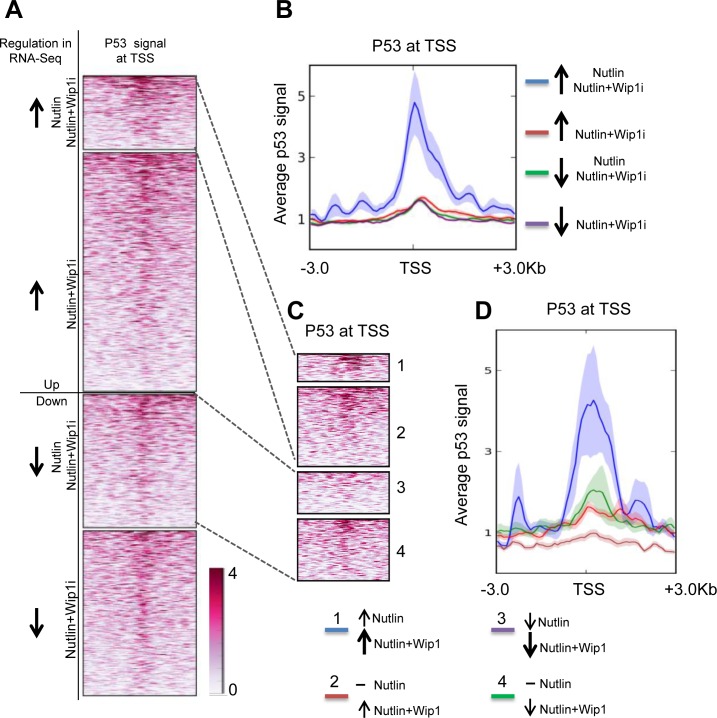
Induction of p53-bound genes by Nutlin and Wip1 inhibition **A.** Heat map of p53 at transcription start sites of genes in MCF-7 cells after Nutlin treatment. Chip data on p53-promoter-associations are displayed, red color reflecting the degree of association with p53. Group 1 indicates those genes which were significantly upregulated by both Nutlin alone and Nutlin+Wip1i treatment in the RNA-Seq analysis from Figure 4. Group 2 indicates those genes that were significantly upregulated upon Nutlin+Wip1i treatment but not by Nutlin alone. Group 3 indicates those genes that were significantly downregulated upon Nutlin and Nutlin+Wip1i and group 4 indicates those genes that were downregulated upon Nutlin+Wip1i treatment but not by Nutlin alone. **B.** Profiler image of p53 occupancy at transcription start sites of genes in MCF-7cells after Nutlin treatment. The profiler image (right) provides the average p53 signal obtained ± 3kb from the transcriptional start site for the genes at each of the above-mentioned conditions. ChIP-seq track data for Nutlin-3a-stimulated MCF-7 cells was obtained from p53 ChIP-Sequencing [40] and downloaded from the Gene Omibus database (ID GSE47043). **C.** Heat map of p53 on the TSSs of genes dependent on both p53 activity and stability. For better evaluation, we distinguished the genes from A, group 1 and 3, into two classifications, i. e. genes that were induced/downregulated further by the combined treatment in comparison to Nutlin alone (1 and 3), and the ones which were already induced/repressed by Nutlin alone to the maximum extent (2 and 4). **D.** Profiler image of p53 occupancy at TSSs of genes dependent on p53 activity and stability. The p53 promoter signal was aggregated along the TSSs of these genes as described in C, and a profiler image is displayed on the right.

When comparing the induced genes with a database of promoters that had been found to associate with p53 in response to Nutlin ([40] Gene Omnibus database ID GSE47043), it turned out that the set of Nutlin-plus-Wip1i-inducible genes was highly enriched for promoter occupation by p53. This enrichment was found to a lesser degree with both of the single drugs, but not in the control-treated cells (Figure 5A and 5B). We furthermore identified p53 promoter binding on genes which were even more responsive towards the combination treatment than single Nutlin treatment. Indeed, we identified an especially strong p53 promoter correlation on these genes, indicating that their transactivation depended on both p53 activity and stability. In comparison, genes that were repressed by Nutlin and further repressed by the combinatorial treatment did not show comparable p53 binding, perhaps reflecting indirect regulation (Figure 5C and 5D).

Taken together, these results indicate that the combination of the two inhibitors induces genes that have p53 associated with their promoters. This notion strongly suggests that the differential gene regulation by the two inhibitors is a direct consequence of the observed p53 activation.

### Combined inhibition of Wip1 and Mdm2 induces cell senescence

**Figure 6:**
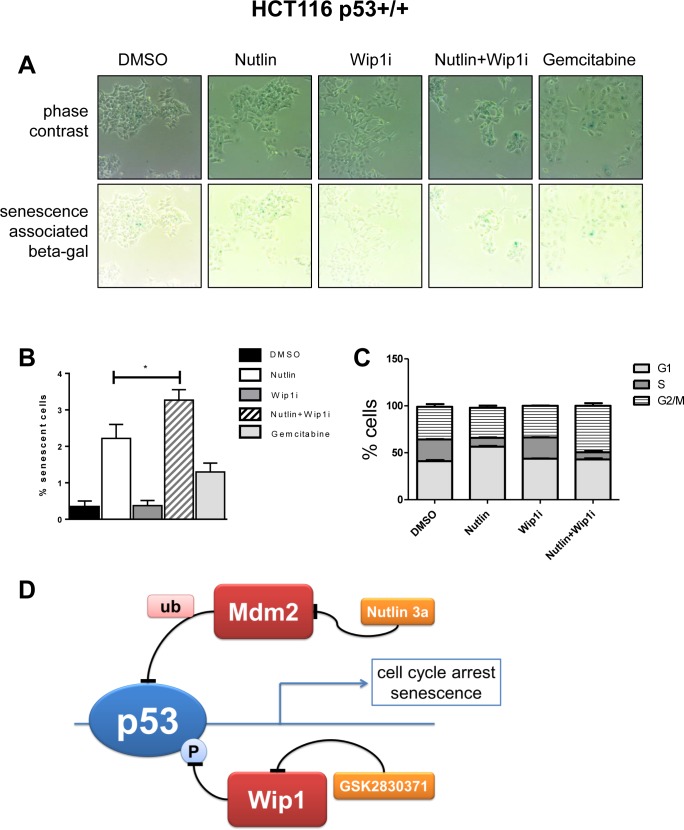
Enhanced cell senescence and G2/M accumulation by the drug combination **A.** Senescence-associated beta-galactosidase (SAB) induced by inhibition of Mdm2 and Wip1. HCT116 p53+/+ were seeded and treated with the indicated combinations of Nutlin and Wip1i (10 μM), or with 30 nM Gemcitabine (positive control), for 48h. Senescent cells were stained using a senescence-associated beta-galactosidase cell staining protocol (Cell Signaling #9860). In the upper row, phase contrast images are provided to visualize all cells, whereas in the lower row, the same areas are shown without contrast, allowing the detection of the blue stain. **B.** Quantification of SAB accumulation. Using bright field microscopy, 10 images under each condition (A) were taken. Quantitative analysis was carried out using ImageJ, and the percentage of senescent cells to total cells was calculated. (mean±SEM). **C.** Flow cytometry to determine the percentage of cells in each phase of the cell cycle. P53-proficient HCT116 cells were treated with the drugs for 24 h and harvested immediately for cell cycle analysis. Using propidium iodide, the percentage of cells in each phase was determined. For the full set of data, cf. Supplemental Figure S4. **D.** Cooperation of Nutlin and Wip1i to enhance p53 activity and cell growth arrest. p53 receives negative feedback upon induction of Mdm2 and Wip1. When both feedback regulators are targeted by drugs simultaneously, p53 activity is enhanced to a greater extent than with each drug alone. As a result, the cells undergo sustainable cell cycle arrest and/or senescence.

Finally, we assessed possible mechanisms of how the combination of Nutlin and Wip1i abolishes cell proliferation. Of note, we had not observed enhanced caspase activity or PARP cleavage (Figure 2 and Supplemental Figure 2), and apoptosis-related pathways were not among the top hits of our GSEA analysis (Figure 4D), arguing against the idea that apoptosis makes a major contribution to drug efficacy in this case. On the other hand, the cells no longer proliferated after combined treatment, even when the drugs had been removed for more than a week (Figure 1A). We therefore suspected that senescence and/or permanent cell cycle arrest was induced upon drug treatment. Senescence was initially described as a mechanism of normal cell aging, due to loss of telomeres. More recently, however, acute senescence was shown to confer the efficacy of chemotherapeutics in many cases [41]. Nutlin induces senescence in a variety of tumor cell lines, albeit to different degrees [42]. We tested whether Wip1i further enhances the amount of senescent cells upon Nutlin treatment. p53-proficient HCT116 cells display senescence-associated beta-galactosidase activity upon treatment with chemotherapeutics [43] and were now assessed as to their senescence response upon treatment with Nutlin and Wip1. Indeed, Nutlin induced senescence in a fraction of cells, but this was further enhanced by Wip1i, whereas the inhibition of Wip1 alone did not lead to a detectable senescence response. Actually, the drug combination exceeded the efficacy of the gemcitabine control in senescence induction (Figure 6A and 6B). Next, we analyzed the cell cycle profile upon treatment with Nutlin, Wip1i and their combination in U2OS cells as well as p53-proficient or p53-deficient HCT116 cells, using propidium iodide (PI) staining and flow cytometry. In p53-proficient cells, Nutlin alone or together with Wip1i reduced the number of cells in S-phase, corresponding to the capability of p53 to induce cell cycle arrest. However, the combination of Wip1i and Nutlin also increased the amount of cells with a 4 n DNA content, corresponding to G2 or M (Figure 6C). This accumulation was dependent on p53 and was still observed at 48 h after removing the drugs (Supplemental Figure S4A-S4D). In conclusion, the combined inhibition of Mdm2 and Wip1 not only induces senescence in a fraction of p53-proficient cells, but also induces a sustainable arrest in G2/M.

## DISCUSSION

Reviving the tumor suppressive activity of p53 has long been attempted for cancer treatment. With the development of Mdm2 inhibitors, this strategy appeared feasible but with limited efficacy. Here we show that the simultaneous inhibition of an additional p53-antagonist, Wip1/PPM1D, further enhances the activity of p53. Combined inhibitors have a considerably greater activity in conferring cell growth arrest, p53 accumulation, and the induction of p53-responsive genes. Thus, the combination of such drugs may provide a stronger anti-cancer treatment efficacy than the sole use of Mdm2-inhibitors (Figure 6D).

Our results agree with and expand recent reports on the use of the Mdm2 inhibitors RG7388 or Nutlin-3 and the Wip1 inhibitor GSK2830371 [29, 30]. In addition to their observations, we provide evidence that inhibition of both targets prevents the outgrowth of cells for ten days (Figure 1), the accumulation of acetylated p53 (Figures 2 and 3), and the finding that each of the inhibitors induces a largely distinct set of genes, whereas the combination of both promotes the enhanced expression of a gene set highly enriched of p53-induced and physically p53-associated genes (Figures 4 and 5).

What makes tumor cells susceptible to the combined treatment? Firstly, a wildtype status for p53 is needed (Figure 3). Furthermore, hyperactive or amplified Wip1 might well render cells more responsive towards Wip1 inhibition. Indeed, MCF-7, U2OS and HCT116 cells all either overexpress Wip1 by means of gene amplification, or carry an activating truncation that removes a regulatory domain from the carboxyterminal portion of the protein [20, 29].

Of note, p53 activation may not always lead to tumor cell killing or permanent cell cycle arrest. On the contrary, we and others have previously observed that p53 activation by Nutlin can have a protective function on cells. Nutlin protects p53-proficient cells against nucleoside analogues and other inducers of replicative stress [44, 45], by temporarily preventing the entry to S phase but perhaps also by regulation of BRCA1 expression [46]. This is generally true with regard to p53-inducing agents [47-50]. Moreover, we have recently shown that Nutlin also provides resistance of cells towards inhibitors of Wee1, a kinase that prevents premature mitosis [51]. Thus, when taking Nutlin and Wip1i to the clinics, care must be taken not to schedule their administration with drugs that require cell cycle progression for their efficacy. Besides nucleoside analogues, antagonists of the mitotic spindle, e. g. taxanes, were shown to be impaired in their efficacy by Nutlin [52], and we anticipate that the same will happen when trying to combine Wip1i, Nutlin, and spindle poisons.

On the other hand, some treatments might further synergize with inhibitors of Wip1 and Mdm2. In particular, it is conceivable that the induction of DNA damage, e. g. through ionizing irradiation, will trigger an ATM-driven response which is no longer counterbalanced by Wip1. This might then further augment the activity of p53 and other pro-apoptotic factors, thereby inducing cell death rather than the mere arrest of cell proliferation. A similar cooperative effect might also be achieved using BH3 mimetics [53] to increase the pro-apoptotic signal at the mitochondria, thereby tipping the balance towards apoptotic cell death.

Finally, interfering with the interaction of p53 and Mdm2 may not provide a block of all Mdm2-induced oncogenic activities. We have recently shown that Mdm2 associates with the polycomb repressor complex 2 (PRC2) and enhances its activities in suppressing gene expression, thus enabling a stem cell phenotype [54]. This activity of Mdm2 is not conferred by its p53-binding region and is thus not detectably affected by Nutlin. To achieve a broader inhibition of Mdm2 in most of its actions, inhibition of the RING finger domain and its E3 ubiquitin ligase activity might be more toxic to cancer cells. Such RING finger inhibitors were described [55], and their combination with Wip1i might yield a more thorough response of tumor cells. Finally, inhibitors that simultaneously interfere with the action of Mdm2 and its heterodimerization partner MdmX/Mdm4 were recently published [56], again showing a broader activity against tumor cells, and may thus warrant combination strategies with Wip1i.

Interfering with Mdm2 to restore the tumor suppressive activity of p53 appears like an attractive but insufficient strategy in most cases. However, the simultaneous interference with additional negative regulators and feedback loops raises the perspective of further boosting p53 and its ability to accumulate, activate transcription, abolish proliferation, and suppress tumor progression.

## MATERIALS AND METHODS

### Cell culture and treatment

U2OS (Osteosarcoma, p53 wild type) and MCF-7 (breast carcinoma, p53 wild type) were maintained in Dulbecco's modified Eagle's medium (DMEM). HCT116 cells (colon cancer, p53 wild type or with a targeted deletion of p53 [32]) were maintained in McCoys 5A medium (1x). Cell culture media were supplemented with 10% fetal bovine serum (FBS) and antibiotics. Cells were maintained at 37°C in a humidified atmosphere with 5% CO_2_. For treatment of cells, Nutlin-3a (Sigma N6287), GSK2830371 (Active Biochem, CAS#:1404456-53-6) stock solutions were prepared in DMSO and then diluted in pre-warmed medium and added to the cells for the indicated periods of time.

### Cell proliferation assay

Cells were seeded in 24-well plates and the treatment was carried out as mentioned. The confluency of the cells was measured using a Celigo cell cytometer (Nexcelom; labeled as Day 0). After 24/48/72 h, the medium was replaced with fresh media; the confluency was determined again (Day 1); subsequent measurements were made every 24 h and media was changed every 48 h.

### Transfection of human cells

Transient transfection of U2OS cells with siRNAs to knock down PPM1D (Ambion silencer select s16288 and s16289, Thermo Fisher), and a corresponding control siRNA was carried out using Lipofectamine 2000. Lipofectamine and siRNA were dissolved separately in DMEM(without FCS, Glutamine, and antibiotics) and incubated at room temperature for 5 min. Later, they were combined and incubated for another 20 min. In one well of a 6-well plate, around 250,000 cells were seeded in 1.5 mL DMEM with supplements, and 500 μl of the Lipofectamine-siRNA mix were added drop-wise. The cells were harvested 48 h after transfection.

### Immunoblot analysis

Cells were harvested in protein lysis buffer (20 mM TRIS-HCl pH 7.5, 150 mM NaCl, 1 mM Na_2_EDTA, 1 mM EGTA, 1 mM beta-glycerophosphate, 2 M Urea, Protease inhibitor Cocktail, Roche). After 10 min lysis on ice, samples were briefly sonicated to disrupt DNA-protein complexes. Total protein concentration was measured using a Pierce BCA Protein assay kit (Thermo Scientific Fisher). After boiling the samples in Laemmli buffer at 95°C for 5 min, equal amounts of protein samples were separated by SDS-PAGE, transferred onto nitrocellulose, and visualized with the following antibodies: PARP1 (9542, Cell Signalling), γH2AX (S139) (9718, Cell Signalling), β-Actin (ab8227 abcam), p21 (2947, Cell signalling), Mdm2 (OP 46, Calbiochem), p53K382Ac (252S, Cell Signalling), p53phosphoS15 (9287S, Cell Signalling), p53 (DO-1 sc-126, Santa Cruz), phospho Chk2 (C13C1 2197, Cell Signalling).

### Determination of drug synergism

Synergism between Nutlin and Wip1i was determined in MCF-7 and U2OS cells. 2000 cells of MCF-7 and U2OS were seeded on 24-well plates. After 24 h, they were treated with the IC 25 and IC 50 concentrations of the individual drugs, Nutlin-3a (Nutlin) and GSK2830371 (Wip1i). For MCF-7 cells, IC 25 and IC 50 for Nutlin was found to be 10μM and 20μM, and IC 25 and IC 50 for Wip1i was 20μM and 40μM. For U2OS cells, IC 25 and IC 50 for Nutlin was found to be 10μM and 20μM, and for Wip1i, IC 25 and IC 50 was 30μM and 40μM. The confluency of cells was measured using a Celigo cell cytometer (Nexcelom). The cell confluency obtained for each drug concentration was normalized to treatment with the DMSO solvent alone. Using the software CompuSyn (www.combosyn.com), the combination index (CI) for the drug combinations was calculated (Chou, Talalay 2010). CI values > 1 describe antagonistic or non-synergistic effects of two drugs, CI = 1 indicates additive effects and CI values < 1 correspond to synergistic effects of combined drug treatment.

### RNA extraction, reverse transcription, and real time quantitative PCR

Total RNA was extracted from cells using TRIzol^®^ (Invitrogen). mRNA was reverse-transcribed using oligo-dT and random hexameric primers, followed by qRT-PCR analysis using SYBR Green (Invitrogen). Gene expression levels were normalized to the mRNA encoding HPRT1, and the analysis was conducted using the ΔΔCt method. qRT-PCR primer sets were chosen as follows:

**Table:** Primer sequences for gene expression studies in human cells

Gene name	Primer sequence
HPRT1	For- ATG CTG AGG ATT TGG AAA GG Rev- TCA TCA CAT CTC GAG CAA GAC
P21	For- CCT GGC ACC TCA CCT GCT CTG CTG Rev- GCA GAA GAT GTA GAG CGG
PUMA	For- GCC AGA TTT GTG AGA CAA GAG G Rev- CAG GCA CCT AAT TGG GCT C
GADD45α	For- TCA GCG CAC GAT CAC TGT C Rev- CCA GCA GGC ACA ACA CCA C
Wip1	For- CTG AAC CTG ACT GAC AGC CC Rev- CTT GGC CAT GGA TCC TCC TC
PIG3	For- GCT TCA AAT GGC AGA AAA GC Rev- GTT CTT GTT GGC CTC CAT GT

### RNA sequencing

For RNA-sequencing, the quality of total RNA was determined using the Bioanalyzer 2100 from Agilent. All samples analyzed exhibited a RNA Integrity Number > 8. Library preparation was conducted using the TruSeq RNA LT SamplePrep Kit (Illumina), starting from 1000 ng of total RNA. Barcodes for sample preparation were used according to the indications given by the protocol. Accurate quantitation of cDNA libraries was performed with the QuantiFluor™dsDNA System (Promega). The size range of final cDNA libraries was determined applying the DNA 1000 chip on the Bioanalyzer 2100, (Agilent; 290-310 bp). cDNA libraries were amplified and sequenced *via* cBot and HiSeq 2000 (Illumina; SR, 1×50 bp, 6 Gb/sample ca. 30 million reads per sample). Sequence images were transformed with Illumina software BaseCaller to bcl files, which were demultiplexed to fastq files with CASAVA (version 1.8.2). Quality check was performed *via* FastQC (version 0.10.1, Babraham Bioinformatics). Fastq files were mapped to the human reference transcriptome (UCSC hg19) using Tophat (Galaxy Version 0.9) [57] Read counts for each sample and each gene were aggregated using a htseq-count [58]. DESeq2 (version 1.10.1) was used for measuring differential expression [59]. RNA library preparation and sequencing was done by the Transcriptome Analysis Laboratory (TAL, Göttingen).

Gene set enrichment analysis (GSEA) from C2 curated gene sets (provided by the Molecular Signatures Database (MSigDB) v5.0) was performed using variance stabilized normalized read counts. [60, 61]. The threshold of significant enrichment (q≤0.25) was implied according to the GSEA standards (http://www.broadinstitute.org/gsea/doc/GSEAUserGuideFrame.html).

### Correlation of RNA-Seq and p53 ChIP-seq data

Raw data for p53 ChIP-Sequencing [40] were downloaded from the Gene Omibus database (ID GSE47043). The reads were mapped to the human reference genome (UCSC hg19) using Bowtie (version 1.0.0) [62]. Peak calling was done by Model-based Analysis of ChIP-Seq (version 1.4.2 [63]. Coverage was determined by normalizing the total number of mapped reads per hundred million. p53 enrichment was analyzed on the transcriptional start sites (TSSs) of genes that were upregulated in MCF-7 cells after Nutlin and after Nutlin+Wip1i treatment using deeptools functions [64] based on the Galaxy framework [65].

### Caspase activity assay

Cells were seeded in 6-well plates and treated with drugs, At 24h post-treatment, cells were harvested (inclusive of medium) and centrifuged at 1500xg for 5 min at 4°C. The pelleted cells were resuspended in 250μl caspase lysis buffer (1M Tris-HCl, 2mM MgCl_2_, 150mM NaCl, 10mM DTT, protease-inhibitor (Roche complete mini)). They were shock-frozen thrice in liquid nitrogen and centrifuged at 15,000xg for 15 min at 4°C. 40μl of lysate was pipetted per well in a 96-well plate in triplicates. 10μl of Ac-DEVD-AMC substrate (working concentration 25μM) (ALX-260-031 Enzo) was added to each sample. Caspase activity was measured using a fluorometer (Synergy MX 267137) at excitation wavelength 380nm and emission wavelength 460nm every 10 min for 4 h at 37°C.

### Cell cycle analysis by flow cytometry

Cells were seeded in 6-well plates and treated with DMSO, Nutlin, Wip1i, and Nutlin+Wip1i. After fixation in ethanol, the cells were washed with 0.05% Triton-X in PBS. Subsequently, the cells were resuspended in 1 mg/ml RNAse A solution in PBS and incubated for 30 min at 37°C, and then with propidium iodide (final concentration: 30 μg/ml). Flow cytometry was performed using the Guava PCA-96 Base System (Millipore), and the percentage of cells in each phase of the cell cycle was determined using the Guava Express Pro software.

## SUPPLEMENTARY MATERIAL FIGURES AND TABLES








