# Supplementary material for: Cooperation of Nutlin-3a and a Wip1 inhibitor to induce p53 activity
Source: Oncotarget. 2016 May 11;7(22):31623–38. doi: 10.18632/oncotarget.9302 (PMC5077964; doi:10.18632/oncotarget.9302)
Supplement: Supplementary file 1 [file oncotarget-07-31623-s001.pdf]

Cooperation of Nutlin-3a and a Wip1 inhibitor to induce p53 activity

Supplementary Material

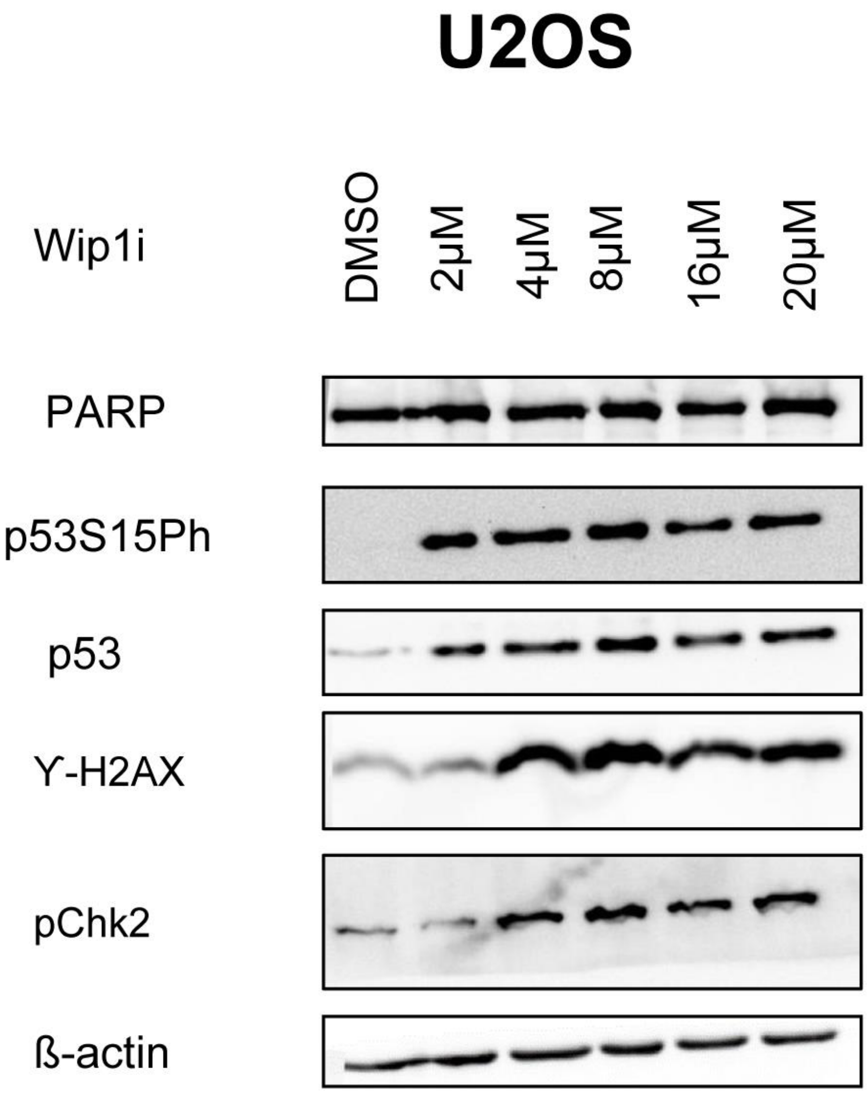

SUPPLEMENTAL FIGURE 1. Wip1i titration in U2OS cells. Related to Fig. 1

U2OS cells were treated with varying concentrations of Wip1i GSK 2830371. After 24 h, the cells were harvested, followed by immunoblot analysis to detect the DNA damage-induced kinase substrates p53, phospho-p53, phospho-H2AX, and phospho-Chk2. PARP staining revealed the absence of detectable caspase activity. Actin staining served as the loading control.

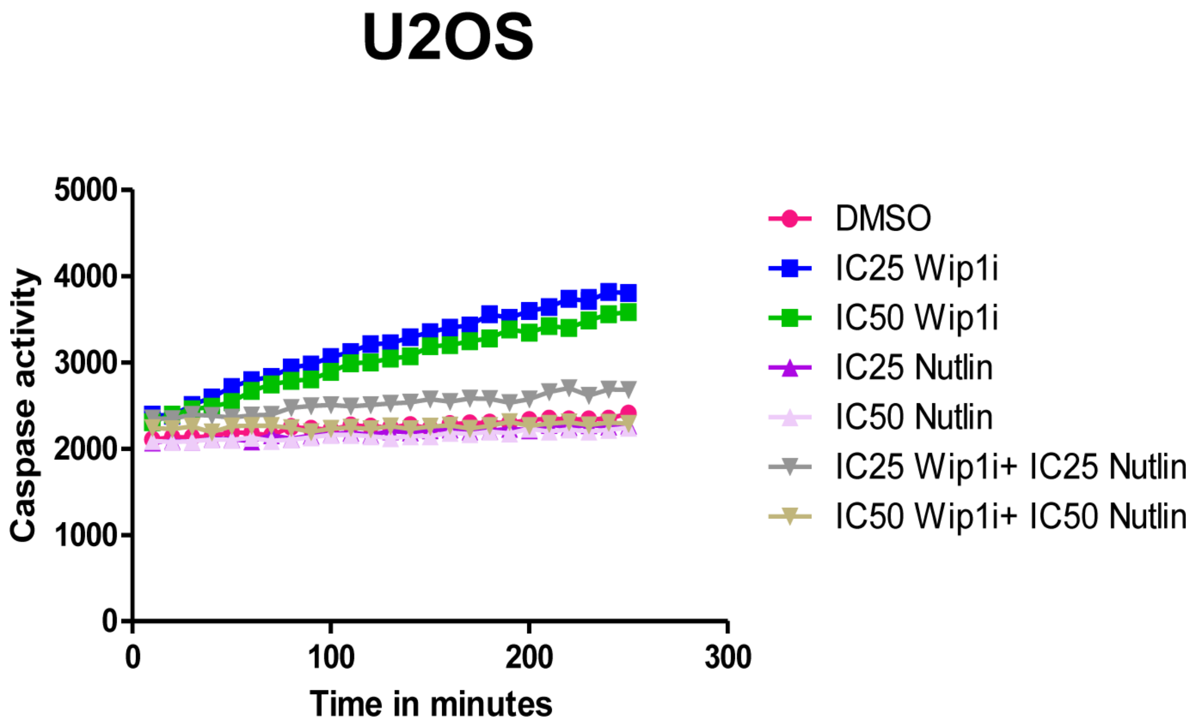

SUPPLEMENTAL FIGURE 2. Lack of caspase activation upon simultaneous inhibition of Mdm2 and Wip1. Related to Fig. 2

U2OS cells were treated as indicated, followed by harvest and lysis to assay for Caspase 3 activity after 24h. A fluorescent substrate to Caspase 3 was added to the lysates, and cleavage was followed over time. Note that Wip1i alone induces a moderate degree of Caspase activation, but Nutlin or the combinations do not. This assay could only be carried out in U2OS cells but not in MCF-7 cells, since the latter are lacking functional caspase 3 [66].

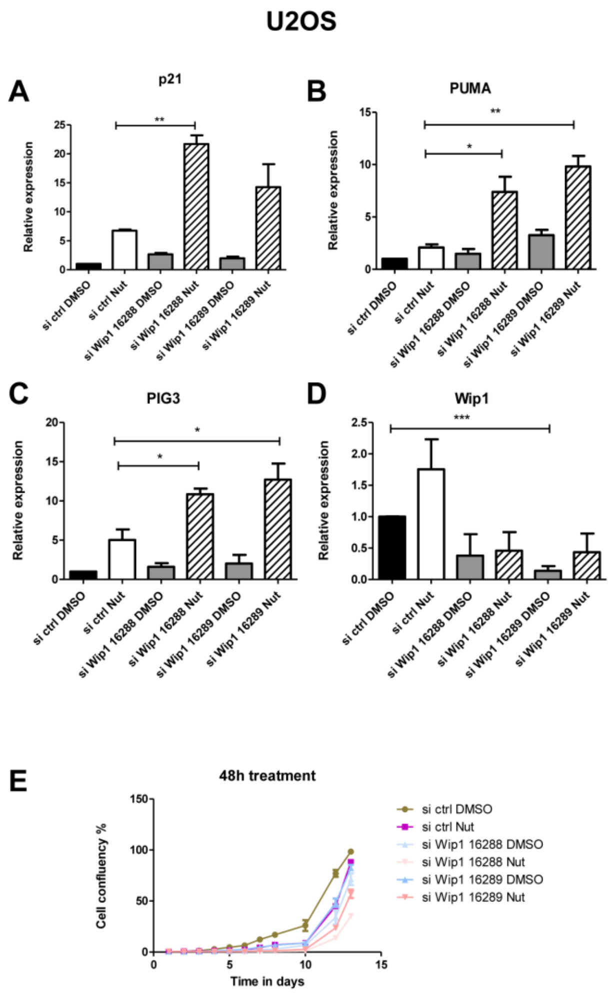

SUPPLEMENTAL FIGURE 3. Wip1 knockdown along with Nutlin treatment leads to the activation of p53 target genes and cooperatively reduces cell proliferation. Related to Fig. 3

siRNA knockdown of Wip1 was combined with Nutlin treatment in U2OS cells. Two sets of siRNA against Wip1 were used.

A-D Quantitative RT-PCR was carried out to quantify the expression of p53 target genes, namely p21, PUMA and PIG3.

E the proliferation of U2OS cells was monitored after Wip1 knockdown and/or Nutlin treatment for 48 h.

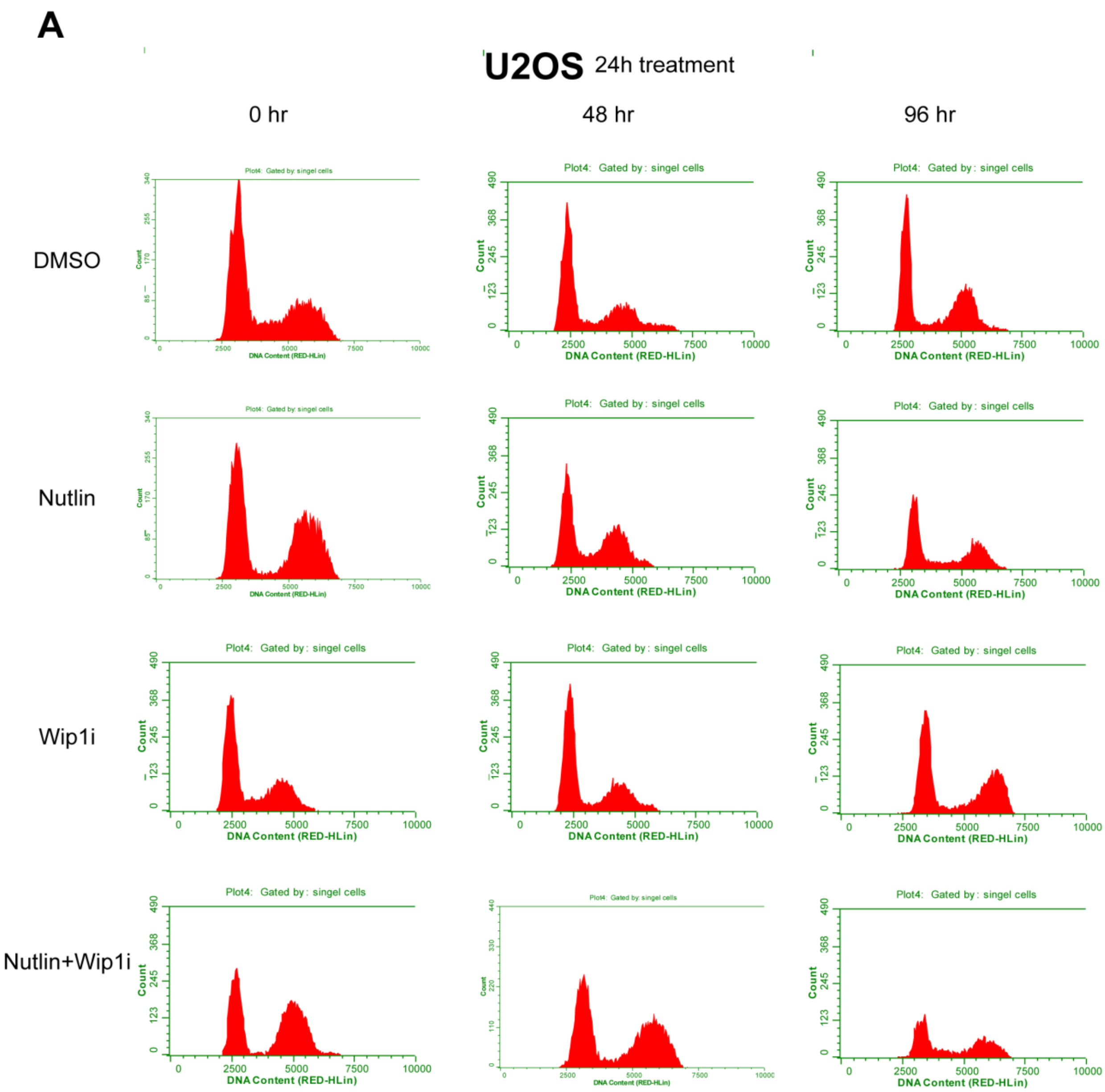
A
U2OS 24h treatment
0 hr
48 hr
96 hr
DMSO
Nutlin
Wip1i
Nutlin+Wip1i
Plot4: Gated by: singel cells
Count
DNA Content (RED-HLin)

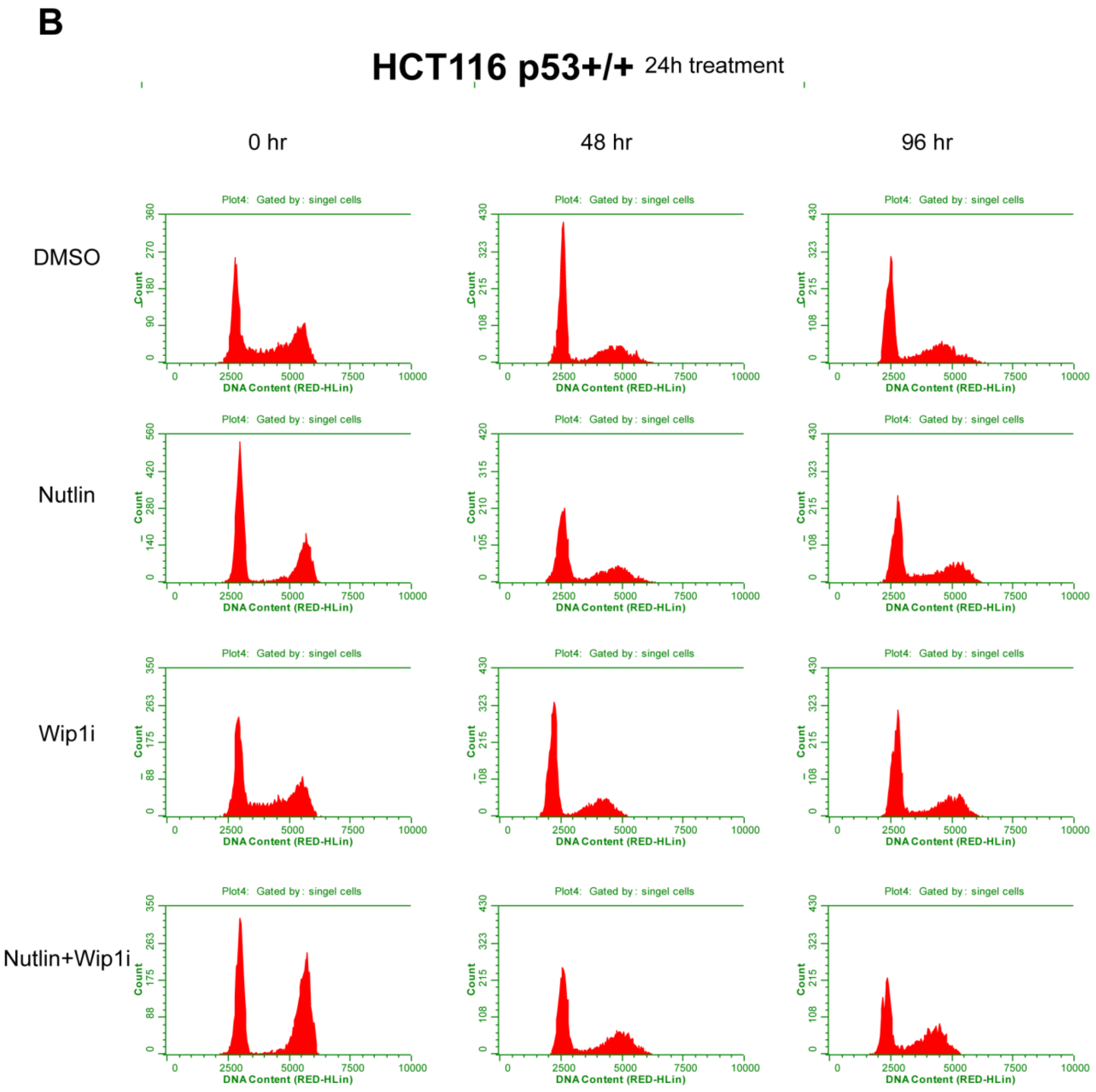

B
HCT116 p53+/+ 24h treatment
0 hr
48 hr
96 hr
DMSO
Nutlin
Wip1i
Nutlin+Wip1i
Plot4: Gated by: singel cells
Count
DNA Content (RED-HLin)
0
2500
5000
7500
10000

C

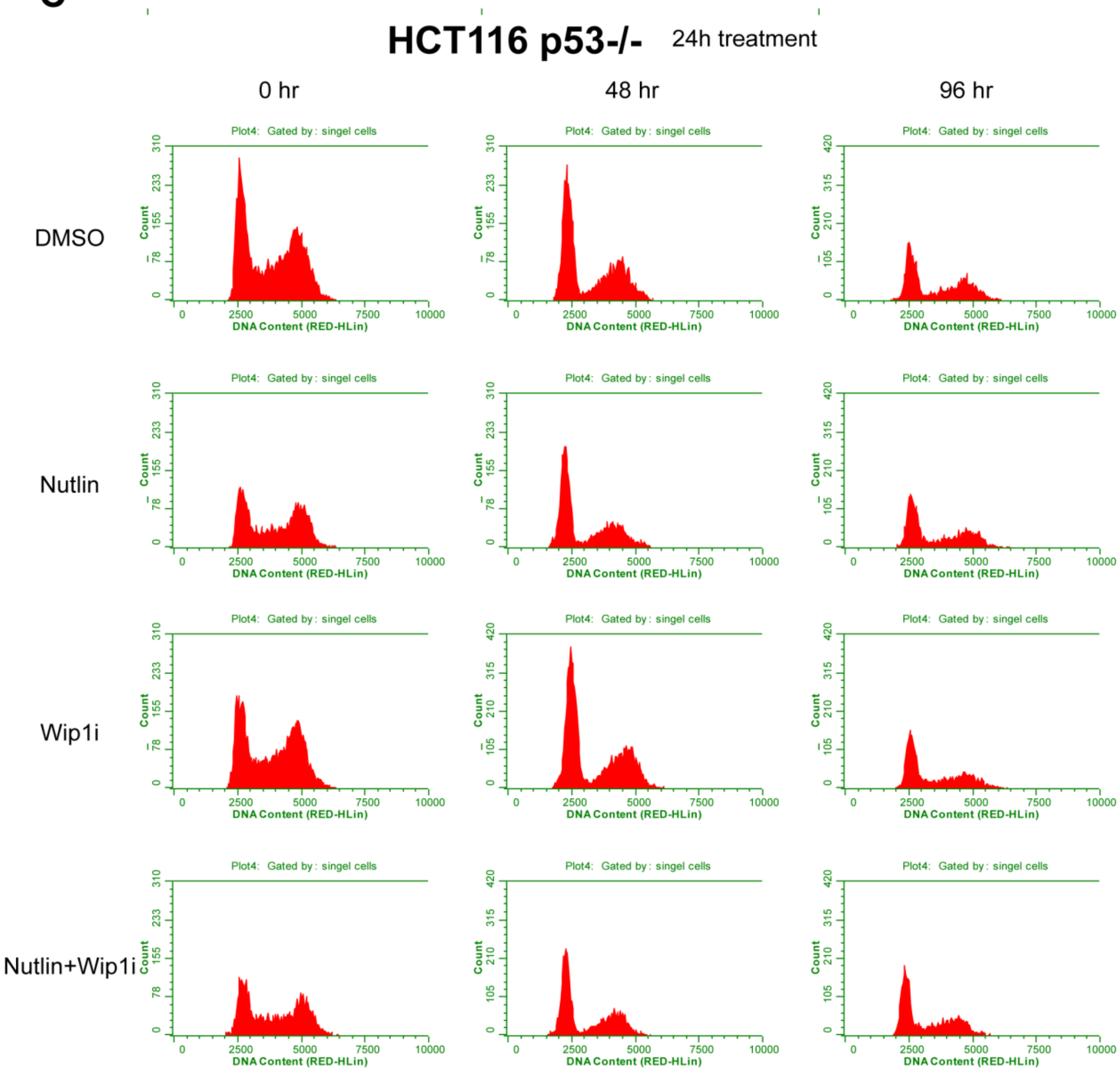
HCT116 p53-/- 24h treatment
0 hr
48 hr
96 hr
DMSO
Nutlin
Wip1i
Nutlin+Wip1i
Plot4: Gated by: singel cells
Count
DNA Content (RED-HLin)
0
2500
5000
7500
10000
78
155
233
310
105
210
315
420

D

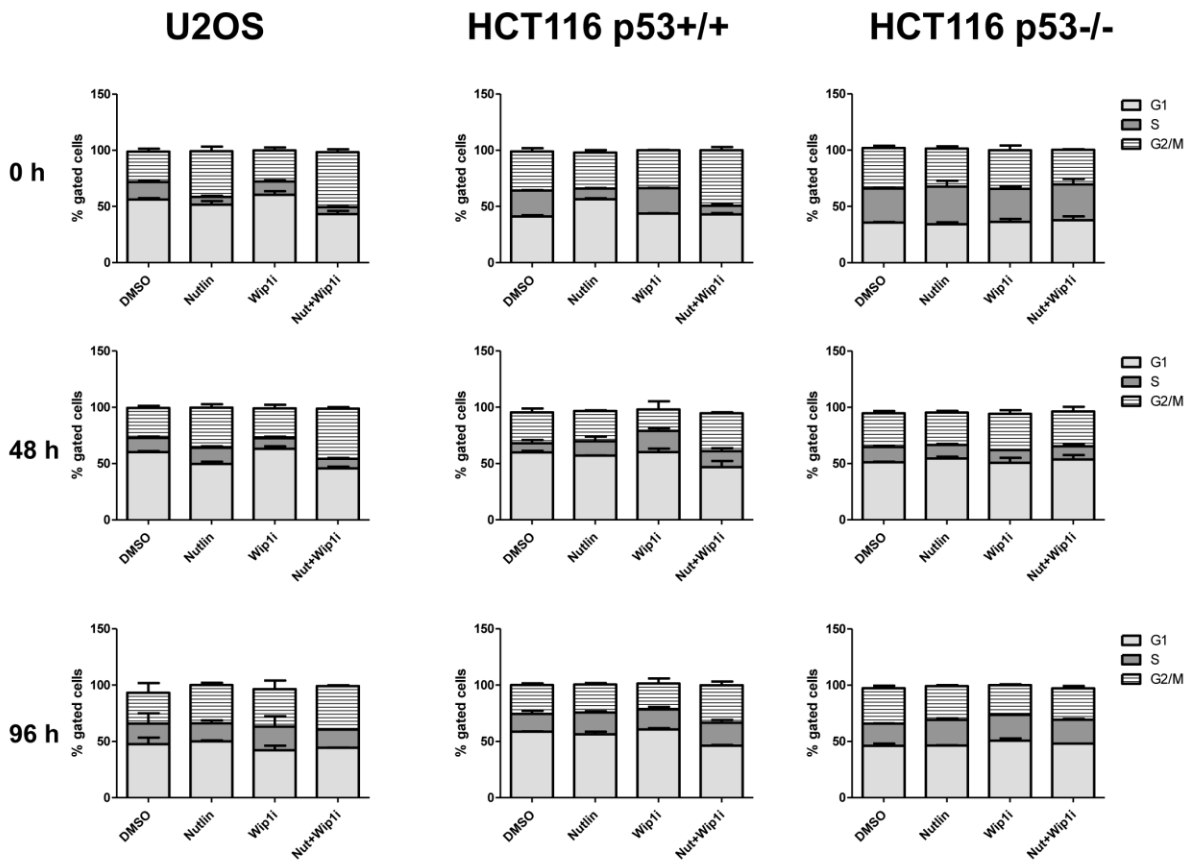

SUPPLEMENTAL FIGURE 4. Cell cycle analysis upon treatment with Nutlin, Wip1i and their combination, at different time points after treatment. Related to Fig. 6

U2OS and HCT116 (p53 proficient or deficient) cells were treated with Nutlin, Wip1i and their combination for 24 h. The cells were then harvested for cell cycle analysis immediately (referred to as 0h), or incubated with fresh medium and harvested after 48h or 96h. The cell cycle profiles were determined by propidium iodide staining and flow cytometry, and are provided along with the percentage of cells in each phase.

Table S1_MCF-7_RNASeq, related to Fig. 4

MCF-7 cells were treated with 10 µM of Nutlin, 10 µM of GSK 2830371 or a combination of both drugs for 16 h. Global gene expression was analyzed via next generation RNA-sequencing (cf. Fig. 4A). Normalized RNA-seq reads were analyzed by DESeq2. Base mean values, log2 fold change (FC) and adjusted p values are shown for each gene.

Table S2 _MCF-7_C2GSEA, related to Fig. 4

Gene set enrichment analysis (GSEA) from C2 curated gene sets (provided by the Molecular Signatures Database (MSigDB) v5.0) was performed using variance stabilized MCF-7 RNA-Seq data from Nutlin and Nutlin+Wip1i treated samples [60, 61]. The threshold of significant enrichment ($q \leq 0.25$) was implied according to the GSEA standards (http://www.broadinstitute.org/gsea/doc/GSEAUserGuideFrame.html).

Table S3 _MCF-7_p53TSS_intensity, related to Fig. 5

p53 enrichment was analyzed on the transcriptional start sites (TSSs) of genes that were regulated in MCF-7 cells after Nutlin and after Nutlin+Wip1i treatment. Tabs 1-4 denote the following regulation patterns: 1) genes upregulated after Nutlin and Nutlin+Wip1i treatment; 2) genes only upregulated after Nutlin+Wip1i treatment; 3) genes downregulated after Nutlin and Nutlin+Wip1i treatment; 4) genes downregulated only upon Nutlin+Wip1i treatment. Raw data for p53 ChIP-Sequencing [39] were downloaded from the Gene Omnibus database (ID GSE47043).
